# Acute lung injury and the acute respiratory distress syndrome in Ireland: a prospective audit of epidemiology and management

**DOI:** 10.1186/cc6808

**Published:** 2008-02-29

**Authors:** The Irish Critical Care Trials Group

**Affiliations:** 1 22 Merrion Square North, Dublin 2, Ireland

## Abstract

**Introduction:**

The aim of this study was to describe the epidemiology and management of acute lung injury (ALI) and the acute respiratory distress syndrome (ARDS) in Ireland.

**Methods:**

As part of a 10-week prospective national audit of patient demographics and organ failure incidence in intensive care in Ireland, all patients with ALI/ARDS in 14 participating centres were prospectively identified using American European Consensus Conference definitions.

**Results:**

There were 1,029 admissions during the study period; of these, 728 patients were invasively ventilated. A total of 196 (19%) patients had ALI/ARDS, and 141 of these (72%) had ALI/ARDS on admission and a further 55 (28%) developed ALI/ARDS after admission. For the patients with ALI/ARDS, the mean (± standard deviation) age was 58 ± 17 years and 62% were male. The most common predisposing risk factors were pneumonia (50%) and nonpulmonary sepsis (26%). Mean (± standard deviation) tidal volume/kg was 7.0 ± 1.7 ml/kg. Median (interquartile range) duration of ventilation was 6.8 (2.0 to 12.8) days. Median (interquartile range) length of stay in the intensive care unit was 10.0 (5.0 to 18.5) days. The overall intensive care unit mortality for ALI/ARDS was 32.3%. Lower baseline arterial oxygen tension/fraction of inspired oxygen ratio and higher Sequential Organ Failure Assessment scores were associated with increased mortality. Although not significant, patients receiving treatment with a statin during admission had a 73% lower odds of death (odds ratio 0.27, 95% confidence interval 0.06 to 1.21; *P *= 0.09).

**Conclusion:**

The incidence of ALI/ARDS is high and is associated with significant mortality. Protective lung ventilation is used commonly throughout participating centres. With low tidal volume ventilation, the degree of hypoxaemia is associated with outcome. These data will inform future multicentre clinical trials in ALI/ARDS in Ireland.

## Introduction

Acute lung injury (ALI) and the acute respiratory distress syndrome (ARDS) occur in response to a variety of insults and are characterized by the development of noncardiogenic pulmonary oedema, impaired gas exchange and need for mechanical ventilation. ALI/ARDS is a major cause of acute respiratory failure associated with significant morbidity and mortality. Delivery of critical care to patients with ALI/ARDS accounts for a significant proportion of intensive care unit (ICU) capacity. The mean cost per ICU bed-day is €2,000 [1] and demand for ICU beds exceeds supply.

Many pharmacological treatments for lung injury have been evaluated, but none have clearly decreased mortality [2]. There is therefore an urgent need to develop novel treatment strategies for patients with ALI/ARDS. Notably, statins – a promising potential new therapeutic option – modulate mechanisms that are important in the development of lung injury [3]. Statins attenuate lung injury *in vivo *in animal models, including ischemia-reperfusion [4], peritonitis [5] and endotoxaemic sepsis [6].

The Irish Critical Care Trials Group (ICCTG) was formed in 2006 with the aim of improving the capacity to conduct high quality clinical research in the critically ill in Ireland. In order to inform hypotheses, feasibility and design of multicentre clinical trials, there was a need to define first the epidemiology of the potential study population. Accordingly, the ICCTG conducted a prospective 10-week national audit of patient demographics and organ failure incidence in intensive care in Ireland.

The purpose of this article is to report on the cohort of patients within the study group with ALI/ARDS in the Irish adult ICU population in order to determine the incidence, aetiologies and mortality of ALI/ARDS; to establish factors associated with outcome; to identify whether standardized care is being delivered across participating centres in a research network; and to investigate whether treatment with statins is beneficial to provide pilot data for subsequent clinical trials.

## Materials and methods

A prospective 10-week national audit of patient demographics and organ failure incidence in intensive care in Ireland was conducted across the 14 general ICUs that form the ICCTG. Local research ethics committee approval was required and granted in nine centres. The need for informed patient consent was waived by the local ethics committees in these centres. Research ethics committee approval was not required at the other centres (in which the project was categorized as audit) because patient management was not altered, only routinely collected data were used and the data were fully anonymized. These centres include a combination of general units of varying size and tertiary referral ICUs, including three ICUs with neurosurgical beds.

As part of this audit, all patients admitted to the ICU were screened daily during their entire admission for the development of ALI/ARDS. Patients were included in the study if they fulfilled the American European Consensus Conference (AECC) criteria [7] for ALI/ARDS (acute onset of bilateral infiltrates on chest radiograph; arterial oxygen tension [PaO_2_]/fraction of inspired oxygen [FiO_2_] ratio <40 kPa for ALI and <27 kPa for ARDS; and absence of cardiac failure or left atrial hypertension [assessed clinically, echocardiographically, or with invasive monitoring]) and required invasive ventilation. Patients were defined as having ALI/ARDS on admission if criteria were fulfilled within 48 hours of admission. If criteria were fulfilled after 48 hours, then patients were categorized as developing ALI/ARDS after admission.

Standard demographic data including individual organ and total Sequential Organ Failure Assessment (SOFA) score [8] were collected daily each morning between 08:00 and 10:00 hours on all patients until ICU discharge. When a patient was identified as having ALI/ARDS, additional data including aetiology of ALI/ARDS, ventilator settings and respiratory variables (including tidal volume [V_t_] normalized to actual body weight [V_t_/kg]), and therapy (including whether the patients received a statin) were recorded. The PaO_2_/FiO_2 _ratio at diagnosis and ventilator settings and respiratory variables (including V_t_/kg and plateau pressure) as well as SOFA score were recorded on the day of diagnosis and then daily each morning between 08:00 and 10:00. Duration of mechanical ventilation and survival status at ICU discharge were recorded.

A form summarizing all ICU admissions and discharges during the previous 24 hours was submitted daily. The total number of ICU admissions determined from the daily report was used to confirm that all patients were included. In addition, this served as a control function to ensure that the participating centres remained active and screened patients throughout the study period. The principal ICU investigator at each centre was responsible for data validation before submission to the coordinating Clinical Research Support Centre (CRSC). Telephone and e-mail assistance from the CRSC was available. The data were entered onto a database at the CRSC and then reviewed for inconsistencies and data entry errors.

### Statistical analysis

Proportions were used as descriptive statistics for categorical variables, mean ± standard deviation for normally distributed continuous variables, and median (interquartile range) was for non-normally distributed continuous variables. Pearson's χ^2 ^test was used to compare categorical variables. To analyze ICU mortality, a backward stepwise logistic regression model was employed to choose from among variables that were associated with mortality and felt to be clinically important. Consequently, age, sex, aetiology, SOFA score, PaO_2_/FiO_2 _ratio, plateau pressure, V_t_/kg, arterial carbon dioxide tension and use of statins were included. *P *< 0.05 was considered statistically significant. All analyses were performed using SPSS version 15.0 (SPSS Inc., Chicago, IL, USA).

## Results

Data were collected on 1,029 admissions during the study period between 7 August and 20 October 2006; of these patients, 728 were mechanically ventilated. A total of 196 (19%) patients had ALI/ARDS, of whom 141 (72%) had ALI/ARDS on admission and a further 55 (28%) patients developed ALI/ARDS after admission. Patient demographics are summarized in Table 1.

**Table 1:** Demographic details in patients with ALI/ARDS

**Parameter**	**Value**
Age (years; mean ± SD)	58 ± 17
Male (*n *[%])	120 (62)
SOFA score on day ALI/ARDS diagnosed (mean ± SD)	8.8 ± 4.1
PaO_2_/FiO_2 _ratio (mean ± SD)	22.7 ± 9.1
Plateau pressure (cmH_2_O; mean ± SD)	21.5 ± 6.8
pH (mean ± SD)	7.34 ± 0.15
PCO_2 _(kPa; mean ± SD)	5.8 ± 1.6
PEEP (cmH_2_O; mean ± SD)	7.7 ± 2.8
Duration of ventilation (days; median [IQR])	6.0 (2.0 to 12.8)
ICU length of stay (days; median [IQR])	10.0 (5.0 to 18.5)

ALI, acute lung injury; ARDS, acute respiratory distress syndrome; FiO_2_, fraction of inspired oxygen; ICU, intensive care unit; IQR, interquartile range; PaO_2_, arterial oxygen tension; PaCO_2_, arterial carbon dioxide tension; PEEP, positive end-expiratory pressure; SD, standard deviation; SOFA, Sequential Organ Failure Assessment.

The overall rate of ICU mortality from ALI/ARDS was 32.3%. There was no significant difference in mortality if ALI/ARDS was present on admission or if it developed after admission (32.8 versus 30.9%, respectively). Patients with ALI had a significantly lower mortality compared with patients with ARDS (21.0 versus 37.8%, respectively; *P *= 0.02).

**Figure 1:**
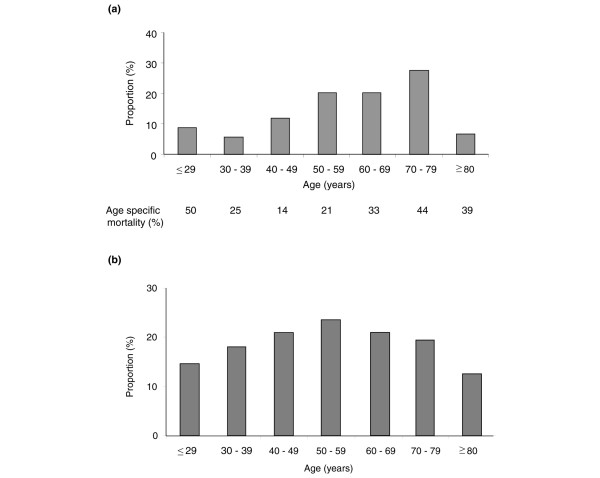
Patients with ALI/ARDS stratified by age and relative to all admissions. **(a) **Proportion of patients with acute lung injury (ALI)/acute respiratory distress syndrome (ARDS) in each age group and **(b) **proportion of patients with ALI/ARDS relative to all admissions in each age group.

Of the patients with ALI/ARDS, a greater proportion were in the older age groups (Figure 1a). However, this merely reflected the age demographics of ICU admissions, because the age-specific incidence was similar in each age group (Figure 1b). There was no significant difference in mortality according to age (Figure 1a).

**Figure 2:**
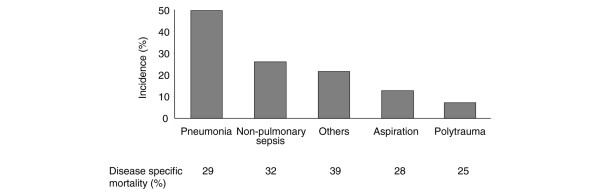
Clinical risk factors for ALI/ARDS and associated risk for mortality. ALI, acute lung injury; ARDS, acute respiratory distress syndrome.

The most common risk factor for ALI/ARDS was pneumonia followed by nonpulmonary sepsis. There was no significant difference in mortality according to risk factor (Figure 2).

**Figure 3:**
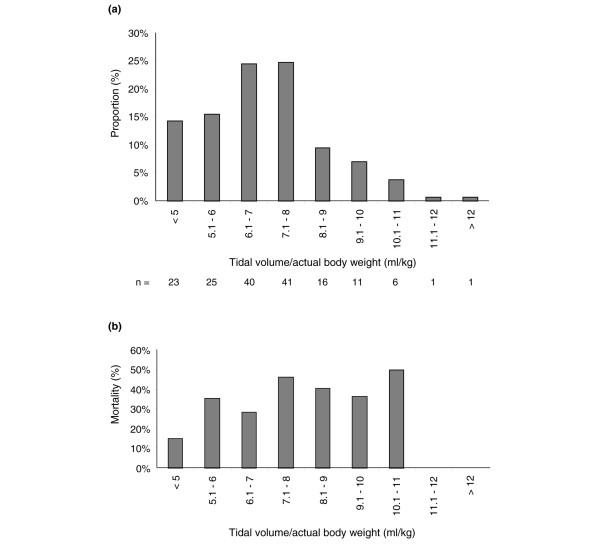
Mean tidal volume. Mean tidal volume (normalized to actual body weight) recorded on the day of diagnosis of acute lung injury/acute respiratory distress syndrome: **(a) **frequency distribution and **(b) **associated mortality.

On the day of diagnosis of ALI/ARDS, patients received mechanical ventilation with a mean V_t _of 7.0 ± 1.7 ml/kg actual body weight (corresponding to approximately 8.4 ± 2.0 ml/kg predicted body weight). Fourteen per cent were ventilated using a V_t _of 5 ml/kg or less (approximately 6 ml/kg predicted body weight), with only 5% ventilated using a V_t _above 10 ml/kg (approximately 12 ml/kg predicted body weight; Figure 3a). Although mortality increased with increasing V_t_, this was not significant (Figure 3b). There was no significant variation between participating centres in V_t_/kg (data not shown). Mean positive end-expiratory pressure (PEEP) was 7.7 ± 2.8 cmH_2_O.

**Figure 4:**
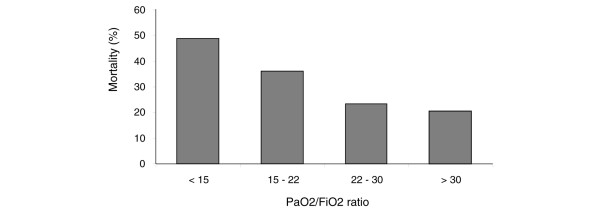
Mortality associated with PaO_2_/FiO_2 _ratio quartiles on the day of diagnosis of ALI/ARDS. ALI, acute lung injury; ARDS, acute respiratory distress syndrome; FiO_2_, fraction of inspired oxygen; PaO_2_, arterial oxygen tension.

**Figure 5:**
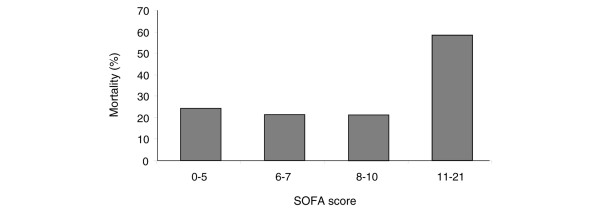
Mortality associated with organ dysfunction on day of diagnosis of ALI/ARDS. Organ dysfunction was assessed based on Sequential Organ Failure Assessment score quartiles. ALI, acute lung injury; ARDS, acute respiratory distress syndrome.

As the PaO_2_/FiO_2 _ratio on the day of diagnosis of ALI/ARDS increased, mortality decreased (*P *= 0.009; Figure 4). ICU mortality was 48.9% for those with a PaO_2_/FiO_2 _ratio under 15 and 20% in those with a PaO_2_/FiO_2 _ratio above 30. There was no significant correlation between V_t_/kg and PaO_2_/FiO_2 _ratio. Mortality was significantly increased in the patients with the highest SOFA score on the day of diagnosis of ALI/ARDS (*P *= 0.001; Figure 5). Only 12% of patients had a plateau pressure above 30 cmH_2_O. Plateau pressure was not associated with mortality. There was no significant correlation between V_t_/kg and plateau pressure. Arterial carbon dioxide tension was not associated with mortality.

**Figure 6:**
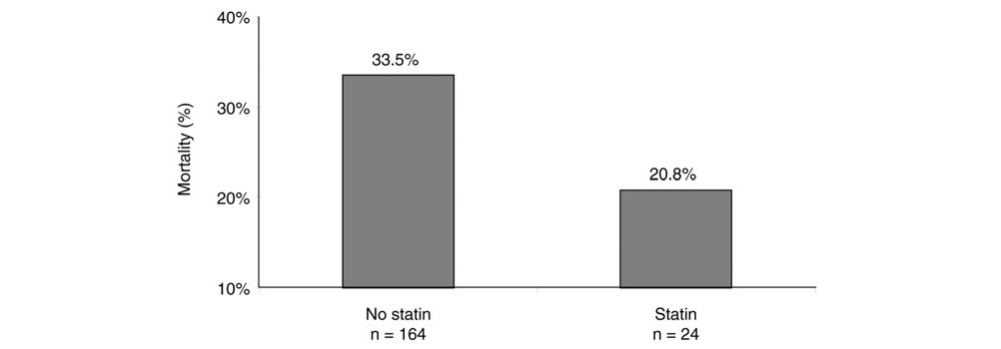
Mortality in patients with ALI/ARDS receiving treatment with a statin during admission. ALI, acute lung injury; ARDS, acute respiratory distress syndrome.

Data on statin use during admission was available for 188 patients. Mortality in the 24 patients who received statins during their ICU stay was reduced compared with mortality in the patients who did not, although this difference was not statstically significant (20.8% versus 33.5%; *P *= 0.2; Figure 6). The demographics and reason for admission of the patients who received statins are shown in Tables 2 and 3, respectively. The patients who received statins were older but there was no other significant difference between patients who received statins and those who did not.

**Table 2:** Demographic details of patients who received a statin

**Parameter**	**Received a statin**	**Did not receive a statin**	***P***
Age (years; mean ± SD)	67.8 (12.4)	56.7 (17.5)	0.01
Male (*n *[%])	13 (56.5)	106 (62.0)	0.61
SOFA score on day ALI/ARDS diagnosed (mean ± SD)	8.1 (3.9)	8.9 (4.1)	0.39
PaO_2_/FiO_2 _ratio (mean ± SD)	20.7 (8.9)	23.0 (9.1)	0.24
Plateau pressure (cmH_2_O; mean ± SD)	23.6 (5.8)	21.2 (6.9)	0.13
pH (mean ± SD)	7.37 (0.10)	7.33 (0.16)	0.24
PCO_2 _(kPa; mean ± SD)	6.0 (1.5)	5.8 (1.6)	0.59
PEEP (cmH_2_O; mean ± SD)	8.3 (3.2)	7.6 (2.7)	0.29

ALI, acute lung injury; ARDS, acute respiratory distress syndrome; FiO_2_, fraction of inspired oxygen; PaO_2_, arterial oxygen tension; PaCO_2_, arterial carbon dioxide tension; PEEP, positive end-expiratory pressure; SD, standard deviation; SOFA, Sequential Organ Failure Assessment.

**Table 3:** Reason for admission in patients who received a statin

**Diagnosis**	**Number of patients**
Pneumonia	7
Nonpulmonary sepsis	4
Respiratory failure	5
Postoperative (AAA repair)	3
ARDS – no aetiology specified	1
Post cardiac arrest	1
Haemorrhagic shock post-IVC filter insertion	1
Neutropenic sepsis, pneumonia, Churg-Strauss syndrome	1
Sarcoidosis	1

In all, 24 patients received statin treatment. AAA, abdominal aortic aneurysm; ARDS, acute respiratory distress syndrome; IVC, inferior vena cava.

Table 4 shows the variables that were independently associated with mortality in the multiple logistic regression model. Only PaO_2_/FiO_2 _ratio and organ dysfunction, as measured using SOFA score, were associated with mortality. Although not significant, patients receiving statins had a 73% lower odds of death (odds ratio 0.27, 95% confidence interval 0.06 to 1.21; *P *= 0.09).

**Table 4:** Multiple regression model on intensive care unit mortality

Variable	OR (95% CI)	p value
PFratio (per 1 unit)	0.91 (0.86–0.97)	0.004
SOFA Score (per point)	1.26 (1.09–1.45)	0.001
Treatment with statins:		
No	Reference	
Yes	0.27 (0.06–1.21)	0.09

CI, confidence interval; OR, odds ratio; SOFA, Sequential Organ Failure Assessment.

## Discussion

In this multicentre survey, we found that ALI/ARDS occurred in approximately 19% of all ICU admissions. Although the incidence is high, it is in keeping with recent well designed epidemiological studies that have estimated the incidence of ALI/ARDS to be 15% to 26% in patients mechanically ventilated for more than 24 hours [9,10]. Because mechanical ventilation was included in our definition of ALI/ARDS, which is not a requirement in the AECC criteria, it is likely that this is a conservative estimate of the incidence of ALI/ARDS.

The crude ICU mortality for ALI/ARDS was lower compared with recent European findings, which estimate the mortality of ARDS at 49% to 53% [9,11]. The mortality rate in this study is in keeping with mortality reported in ARDSnet studies that have utilized protective lung ventilation [12-14]. The lower mortality in this study may reflect the use of a lower V_t _strategy. The mean V_t _in this study was 7.0 ml/kg (approximately 8.4 ml/kg predicted body weight), as compared with a mean V_t _in the European ALIVE study of 8.3 ml/kg (approximately 10.0 ml/kg predicted body weight) [9] and 9.2 ml/kg (approximately 11.0 ml/kg predicted body weight) in a Scottish study [11]. These data suggest advances in the ventilatory management of ALI/ARDS have translated into practice, which is contrast to evidence that many centres still do not apply protective lung ventilation strategies [15,16].

Consistent with previous studies, organ dysfunction predicts mortality in ALI/ARDS [9,17,18]. We found the PaO_2_/FiO_2 _ratio at diagnosis of ALI/ARDS to be associated with mortality. This is contrary to most [19-21] but not all [9] previous reports in ALI/ARDS. The reports demonstrating no association with PaO_2_/FiO_2 _ratio are older and predate the era of protective lung ventilation. With low V_t _ventilation the aim of mechanical ventilation is limitation of ventilator-associated lung injury rather than correction of hypoxaemia. As a result, oxygenation may be more likely to reflect severity of pulmonary dysfunction and outcome. This is supported by the finding there is no correlation between V_t_/kg and PaO_2_/FiO_2 _ratio at diagnosis of ALI/ARDS, which suggests that clinicians do not react to worse oxygenation by using higher V_t_s. It is also important to consider that the PaO_2_/FiO_2 _ratio is highly variable, depending on the ventilatory strategy employed as well as the distribution of ventilation-perfusion, which is influenced by the pattern of mechanical ventilation (including PEEP and I:E ratio) [22]. It is relevant that the initial PaO_2_/FiO_2 _ratio was predictive of outcome in several large clinical trials that implemented a protocolized ventilatory strategy [12,23]. In the present study, although ventilatory strategy was not protocolized, and in particular PEEP/FiO_2 _combinations were not mandated, one possible explanation for the finding that the initial PaO_2_/FiO_2 _ratio was associated with mortality is that ventilatory strategy was sufficiently standardized, which is supported by the close adherence to protective lung ventilation strategy. However, it should also be recognized that physiological indices such as cardiac output may also affect the PaO_2_/FiO_2 _ratio and may have influenced the relationship between PaO_2_/FiO_2 _ratio and mortality. Finally, it is acknowledged that these data relate to PaO_2_/FiO_2 _ratio and V_t _on the day of diagnosis of ALI/ARDS, and the influence of any subsequent alteration in V_t _on the relationship between P/F ratio and mortality is unknown.

V_t _was not associated with mortality in this study, which is not surprising given the relatively good adherence to lung protective ventilation, with only 5% of patients of patients receiving a tidal volume above 10 ml/kg. This is in accordance with previous trials of protective lung ventilation, in which benefit was only seen where there was the largest difference between the lower and higher V_t _strategies [12,24].

Plateau pressure was not related to mortality in this study, in contrast to data describing a direct relationship between plateau pressure and mortality [25,26]. Similarly to the contention that there is no relationship with V_t_, this may reflect adherence to lung protective ventilation, with only 12% of patients having a plateau pressure above 30 cmH_2_O. Furthermore, because plateau pressure is determined by multiple factors, including V_t _(with possible harmful effects as V_t _increases) and PEEP (with possible beneficial effects as PEEP increases [26]), it is possible that this finding reflects opposing influences of V_t _and PEEP in lung injury. We did not define how plateau pressure was to be measured. In addition, 75% of patients received pressure-controlled ventilation, in which inspiratory pressures may overestimate plateau pressure. It is possible that these factors influenced the relationship between plateau pressure and mortality.

Contrary to our findings, age [9,11,17] and duration of ventilation pre-ALI/ARDS [17] have been associated with increased mortality in ALI/ARDS. It is possible that as a result of selection bias elderly patients who were admitted to ICU were less severely ill and had less co-morbidity, which confounded the correlation of age with mortality in our study. Although in some studies risk factors for ALI were found to be associated with mortality [19,27], this has not been a universal finding [9,28], and no such relationship existed in our study. It is more likely that the severity of physiological derangement and organ dysfunction, as in our findings, are more important determinants of outcome.

Accurate epidemiological data are essential to provide pilot data as well as to inform the design and feasibility assessment of clinical trials. These prospective data from an unselected cohort of critically ill patients with ALI/ARDS provide important information to inform phase III clinical trials.

In a study in which mortality is the primary outcome, overestimating the mortality rate in the power calculation will result in the study being under-powered. If the mortality rate of 49% from European epidemiological data [9] were used to determine the required sample size for an ALI/ARDS study in Ireland, where the mortality is lower, this would result in a large and expensive phase III study being significantly under-powered. Another important consideration that may result in overestimation of the current mortality rate for ALI/ARDS is that the European ALIVE study [9] was undertaken in 1999, before the publication of definitive evidence regarding the role of low V_t _ventilation, and subsequent studies showing low adherence to protective lung ventilation [11,15,16] were undertaken relatively soon after publication of the ARDSnet study findings that confirmed benefit. With more widespread adherence to protective lung ventilation, as suggested in this current study, it might be expected that mortality is lower. This has important implications for multicentre studies being performed across Europe.

Furthermore, if the expected mortality benefit from pilot data is small, then powering a study with mortality as the primary outcome may not be possible, because the sample size would be too large to allow recruitment within an acceptable time period. On the basis of such factors, it may be necessary to consider other important clinical end-points such as ventilator-free days in order to design an feasible trial. In addition, these data will inform the decision regarding whether a clinical study could successfully be undertaken nationally or would require multinational trial group co-operation in order to recruit the numbers required.

In undertaking multicentre trials it is essential to demonstrate standardized care between centres. It is reassuring that in this unselected population the standardization of ventilatory strategy was already apparent, with use of lower V_t_s, and that outcomes are in keeping with international standards [13,14].

There is experimental and preclinical evidence that statins [3] may be beneficial in ALI/ARDS. The present study supports an association with improved outcome in patients who received statin therapy. The patients who received statins reflected the general population included in this study. These data are interesting and valuable in terms of generating hypotheses. However, it must be emphasized that the numbers of patients receiving statins were small and that subsequent randomised clinical trials examining statins in ALI/ARDS will be required. A major limitation is that no information was collected on the specific statin or dose administered, which may be important in determining possible benefit. In addition, no information is available on how many patients not receiving statins during their ICU admission had received statins before admission. A further confounding issue is that sicker patients would perhaps be less likely to receive their statin because of potential concerns about increased toxicity or failure of the enteral route in this group.

There are a number of limitations to our study. With the diagnosis of ALI/ARDS based on AECC criteria, it is recognized that misclassification may occur because of misinterpretation of chest radiographs and exclusion of left atrial hypertension on clinical assessment only. However, misclassification is no more likely in this study than in similar epidemiological studies using these current definitions. Data on co-morbidities were not collected. The influence of co-morbidities on overall mortality as well as the potential effect of statins may be important, but this cannot be quantified from the data presented here. Additionally, height was not recorded, and therefore it is not possible to calculate predicted body weight accurately to adjust V_t_. For comparison with the ARDSnet protective lung ventilation study actual body weight has been estimated at 20% greater than predicted body weight, and approximate values for V_t_/predicted body weight have been presented [12]. Data were collected over a 10-week period and seasonal variation in the incidence of ALI/ARDS, although unlikely, cannot be excluded.

## Conclusion

The incidence of ALI/ARDS is high and is associated with significant mortality. Protective lung ventilation, which is known to be associated with lower mortality, is used commonly. There is standardized care throughout participating centres. The high mortality associated with the condition emphasizes the need for clinical trials in ALI/ARDS. These data will help to inform the design of subsequent multicentre clinical trials in ALI/ARDS.

## Key messages

• The use of lower V_t _ventilation in ALI/ARDS suggests advances in ventilatory management are translating into clinical practice.

• Variation in the epidemiology and management of ALI/ARDS should be considered in the design of large national and international randomized clinical studies.

• With the use of protective lung ventilation, severity of impaired oxygenation is associated with outcome from ALI/ARDS.

• Treatment with a statin was associated with a trend toward a reduction in mortality from ALI/ARDS.

## Abbreviations

AECC = American European Consensus Conference; ALI = acute lung injury; ARDS = acute respiratory distress syndrome; CRSC = Clinical Research Support Centre; FiO_2 _= fraction of inspired oxygen; ICCTG = Irish Critical Care Trials Group; ICU = intensive care unit; PaO_2 _= arterial oxygen tension; PEEP = positive end-expiratory pressure; SOFA = Sequential Organ Failure Assessment; V_t _= tidal volume.

## Competing interests

The authors declare that they have no competing interests.

## Authors' contributions

All members of the ICCTG developed the study protocol. DMcA and JW (member of the CRSC) analyzed the data. DMcA wrote the initial manuscript draft. All members of the ICCTG provided critiques of successive drafts of the manuscript. All members of the ICCTG read and approved the final manuscript.

The Irish Critical Care Trials Group are as follows: M Sheridan: Altnagelvin Hospital, M Donnelly: AMNCH Tallaght Hospital, R Bailie: Antrim Area Hospital, M Power: Beaumont Hospital, P Seigne: Cork University Hospital, S Austin: Mater Hospital, Belfast, B Marsh: Mater Miscericordiae University Hospital, C Motherway: Mid Western Region Hospital, M Scully: Our Lady of Lourdes Hospital, C Fagan: St James's Hospital, P Benson: St Vincent's Hospital, D McAuley: Royal Victoria Hospital, J Trinder: Ulster Hospital, J Bates: Galway University Hospitals, K Bailie: Clinical Research Support Centre.
